# What is the phylogenetic signal limit from mitogenomes? The reconciliation between mitochondrial and nuclear data in the Insecta class phylogeny

**DOI:** 10.1186/1471-2148-11-315

**Published:** 2011-10-27

**Authors:** Gerard Talavera, Roger Vila

**Affiliations:** 1 Institut de Biologia Evolutiva (CSIC-UPF), Pg. Marítim de la Barceloneta 37, 08003 Barcelona, Spain; 2 Departament de Genètica i Microbiologia, Universitat Autònoma de Barcelona, Edifici C, 08193 Bellaterra, Spain

## Abstract

**Background:**

Efforts to solve higher-level evolutionary relationships within the class Insecta by using mitochondrial genomic data are hindered due to fast sequence evolution of several groups, most notably Hymenoptera, Strepsiptera, Phthiraptera, Hemiptera and Thysanoptera. Accelerated rates of substitution on their sequences have been shown to have negative consequences in phylogenetic inference. In this study, we tested several methodological approaches to recover phylogenetic signal from whole mitochondrial genomes. As a model, we used two classical problems in insect phylogenetics: The relationships within Paraneoptera and within Holometabola. Moreover, we assessed the mitochondrial phylogenetic signal limits in the deeper Eumetabola dataset, and we studied the contribution of individual genes.

**Results:**

Long-branch attraction (LBA) artefacts were detected in all the datasets. Methods using Bayesian inference outperformed maximum likelihood approaches, and LBA was avoided in Paraneoptera and Holometabola when using protein sequences and the site-heterogeneous mixture model CAT. The better performance of this method was evidenced by resulting topologies matching generally accepted hypotheses based on nuclear and/or morphological data, and was confirmed by cross-validation and simulation analyses. Using the CAT model, the order Strepsiptera was recovered as sister to Coleoptera for the first time using mitochondrial sequences, in agreement with recent results based on large nuclear and morphological datasets. Also the Hymenoptera-Mecopterida association was obtained, leaving Coleoptera and Strepsiptera as the basal groups of the holometabolan insects, which coincides with one of the two main competing hypotheses. For the Paraneroptera, the currently accepted non-monophyly of Homoptera was documented as a phylogenetic novelty for mitochondrial data. However, results were not satisfactory when exploring the entire Eumetabola, revealing the limits of the phylogenetic signal that can be extracted from Insecta mitogenomes. Based on the combined use of the five best topology-performing genes we obtained comparable results to whole mitogenomes, highlighting the important role of data quality.

**Conclusion:**

We show for the first time that mitogenomic data agrees with nuclear and morphological data for several of the most controversial insect evolutionary relationships, adding a new independent source of evidence to study relationships among insect orders. We propose that deeper divergences cannot be inferred with the current available methods due to sequence saturation and compositional bias inconsistencies. Our exploratory analysis indicates that the CAT model is the best dealing with LBA and it could be useful for other groups and datasets with similar phylogenetic difficulties.

## Background

From the seminal comprehensive study of Hennig [1], to the impressive descriptive work of Kristensen [2,3], to the increasingly common molecular approaches [4-14], Insecta class systematics has been a challenging field of study. Molecular phylogenies have become a powerful tool that shed light on many parts of the Tree of Life. At the same time, due to the increasing number of sequences and genomes published, methodological questions are broadly explored by researchers in order to correctly and fully infer evolutionary relationships and patterns. In fact, it is widely accepted that many factors can influence final tree topologies, not to mention supports. Among these factors, we can cite 1) the quality of the sequences and the alignment; 2) the amount of phylogenetic information present in the sequences; 3) the presence of evolutionary biases that are not taken into account by most used evolutionary models (compositional heterogeneity, heterotachy...); 4) the use of markers whose evolution does not reflect the species evolutionary history (paralogs, xenologs); 5) the accuracy of the evolutionary model and the efficiency of the tree search algorithm used for the study [15-19]. Thus, different strategies in the analyses can often lead us to arrive at mutually contradictory conclusions starting from the same dataset. This seems to be particularly true when comparing the studies of relationships among the main taxonomic groups of Arthropoda [20-26]. Intra- and inter-ordinal insect relationships are not an exception and represent a ceaseless source of debate. They have been commonly explored using different types of molecular data: rDNA 18S and 28S, mitochondrial genes, complete mitochondrial genomes, nuclear protein coding genes, the presence of shared intron positions [12] or mitochondrial gene rearrangements [27]. Among the most controversial insect groups with regard to systematic position we can mention the Strepsiptera, an order of obligate endoparasitic and morphologically derived insects. The most basal relationships within the holometabolous and the paraneopteran insects are another example of long-debated relationships.

Mitochondrial genomes have been successful in recovering intra-ordinal phylogenetic relationships concordant with other sources of data, with convincing levels of support, such as in Diptera [28], Hymenoptera [29], Orthoptera [30] and Nepomorpha (Heteroptera) [31]. Nevertheless, mitogenomes proved so far to be generally inadequate to study inter-ordinal relationships of insects and deeper levels of Arthropoda, frequently resulting in strong incongruence with morphological and nuclear data, poor statistical supports, and high levels of inconsistency among different methods [16,24-26,32]. Indeed, comparative studies that contrast nuclear and mitochondrial datasets suggest that nuclear markers are better suited to deal with deep arthropod relationships, as the mitochondrial genome is on average more saturated, biased, and generally evolves at a much faster rate than the nuclear genome [33,34]. Thus, knowing the specific limits for each set of mitogenomes analyzed, i.e. when substitution rates result in saturation that distorts the phylogenetic signal at deeper nodes, is crucial to assess their usefulness in phylogenetics [35].

It is well known that arthropod mitochondrial genomes present some anomalous characteristics, like very high percentage of AT content, frequent gene rearrangements [36] or accelerated evolutionary rates likely related to phenotypic changes in body size or to parasitic lifestyle [37], all of which can limit their applicability in phylogenetic reconstruction. These biases in the data can result in systematic errors when the evolutionary model used for phylogenetic inference does not take them into account. Thus, homogeneous models of substitution or replacement where all sites evolve under the same substitution process [38] and constantly through time [19,39] are not adequate for Arthropoda. One of the most usual artefacts, especially in deep relationships where mutational saturation exists [40], is the long-branch attraction (LBA), a systematic error where two or more branches tend to cluster together producing false relationships [41]. Also, models not accounting for heterogeneity in nucleotide composition among taxa [16] can lead to artefactually group unrelated taxa with similar base composition [42-45].

For all these reasons, artropods in general and insects in particular, constitute an excellent model to tackle challenging questions of phylogenetic methodological interest. Several strategies have been designed to minimize potential biases: 1) Increasing the taxon sampling as far as possible, although generally counteracted by the removal of taxa with an evidently incorrect placement disturbing the reconstruction. 2) Filtering genes in large phylogenomic analyses to avoid paralogy problems and unexpected effects of missing data [45-47]. 3) The use of more specific substitution/replacement models. For example, matrices of amino acid replacement have been designed for Arthropoda (MtArt) [48,49] and Pancrustacea (MtPan) [25]. 4) Removing fast-evolving sites according to discrete gamma category [40,50-52]. 5) Removing third codon position or recoding them as purines and pyrimidines (RY-coding) in DNA alignments [23,53] to reduce the effects of saturation. 6) Using a site-heterogeneous mixture model (CAT) to allow flexible probabilities of the aminoacid replacement equilibrium frequencies, in order to minimise LBA effects [38,54,55].

In this work, we test the performance of different phylogenetic methodological strategies, using mitochondrial genomes of the Class Insecta as a model and including long-branched problematic taxa within Hymenoptera, Strepsiptera, Thysanoptera and Phthiraptera orders that have been usually excluded from mitochondrial datasets. We address controversial taxonomical questions at three different levels of divergence, for which solid hypotheses based on nuclear phylogenies and morphological data exist. Our results show strong differences among the methods tested in their power to resolve inter-ordinal relationships. Using both real and simulated data (see Additional file 1), we confirm the capacity of the site-heterogeneous mixture model (CAT) under a Bayesian framework, currently implemented in software PhyloBayes [38,56], to substantially avoid the LBA artefacts. We show for the first time that the reconciliation between mitochondrial and previous nuclear and morphological knowledge is possible in the cases studied.

## Results and Discussion

### About the exploratory phylogenetic framework

**Figure 1:**
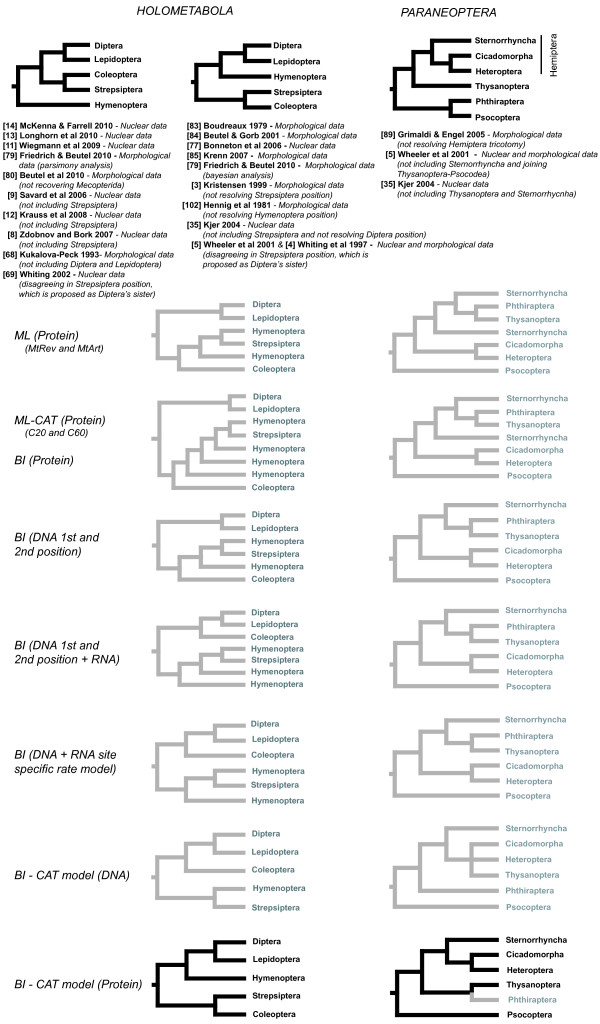
**Phylogenetic strategies tested**. Current knowledge on main holometabolan (two competing hypotheses) and paraneopteran relationships, based on nuclear and morphological data. Topologies obtained with the phylogenetic strategies tested are represented below (relationships matching the currently accepted hypotheses are highlighted in black)

After applying a variety strategies for phylogenetic inference, we compared the trees obtained to the most widely accepted nuclear DNA and morphology-based hypotheses for Holometabola and Paraneoptera systematics (Figure 1). These hypotheses were carefully selected from bibliography based on a great variety of data sources. As a result, we grouped the proposed relationships within Holometabola in two main hypotheses mainly disagreeing in the position of the order Hymenoptera, and a single general hypothesis for Paraneoptera, although this group has been much less intensely studied.

Methodologies that produced topologies identical or very similar to these hypotheses were considered better than those that resulted in very different trees. We noticed a strong susceptibility of our data to the type of analyses performed, confirming once more the instability of phylogenies based on insect mitochondrial genomes. Indeed, almost each approach resulted in a different topology and only the Bayesian inference using the CAT model with amino acid sequences (BI-AA-CAT) was able to obtain trees fitting potentially correct hypotheses. A better performance of the CAT model was confirmed using simulations (Additional file 1; Figure S1). No differences were observed between the MtRev and MtArt models in ML trees for any dataset. Cross-Validation statistics were performed to test the fit of the replacement models for proteins MtRev and CAT to the data. A better fit of the CAT model for Paraneoptera (mean score = 9.216 ± 12.038) and Eumetabola (mean score = 5.75, SD = ± 32.3539), and a similar fit for Holometabola (mean score = -0.055, SD = ± 32.3539) were detected.

The site-heterogeneous mixture model CAT [38] assumes the existence of distinct substitution processes, which usually results in a better fit to the data than site-homogeneous models based on empirical frequencies of amino acid or nucleotide substitutions, like MtRev or GTR [57-60]. In fact it has already been shown in other taxonomical groups that the CAT model is very powerful to overcome LBA artefacts [45,54,61-63]. Thus, the use of models accounting for compositional heterogeneity in the replacement process seems to be more effective than strategies focused on the removal of saturated positions in the case of Insect mitogenomes. Combining the CAT model with the use of amino acid sequences instead of DNA, which should reduce saturation biases, under a Bayesian framework produced the most satisfactory results. The topologies resulting from the analyses with different methods are discussed further on.

### Holometabola phylogeny and the Strepsiptera problem

**Figure 2:**
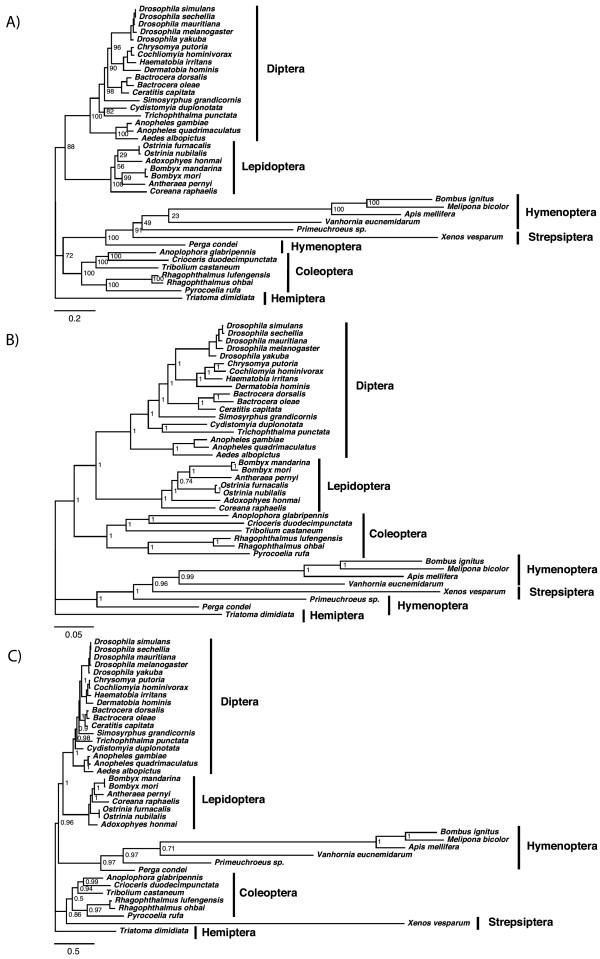
**Holometabola phylogenies**. Holometabola phylogeny using A) ML-AA (2731 positions), B) BI-DNA (9536 positions) and C) BI-AA-CAT (2731 positions). Values at nodes show bootstrap or posterior probabilities, and scale bar represents substitutions/site.

In our dataset for the Holometabola we included one strepsipteran and taxa of the Hymenoptera usually removed from mitochondrial analyses because of their long branches. We observed strong discrepancies among the methodologies used, ML-AA (Figure 2A), BI-DNA (Figure 2B) and BI-AA-CAT (Figure 2C) (see methods for details), confirming the difficulties introduced by such groups. The Strepsiptera species *Xenos vesparum *[64] appeared within Hymenoptera in the ML-AA tree, being completely trapped by the longest branches of the hymenopterans. The same happened with BI-DNA, although in that case, *Xenos *appeared in a more basal position within the hymenopterans, apparently slightly reducing the LBA effect. Finally when applying the BI-AA with the CAT model the LBA was suppressed, revealing completely different positions for the very long branches of Hymenoptera clade and the Strepsiptera (Figure 2C). The topology obtained in this case indicated a sister group relationship between Strepsiptera and Coleoptera (the composite clade being known as Coleopterida), and supported Diptera + Lepidoptera (Mecopterida), which represents the first evidence that mitochondrial data supports these groups.

Since their discovery, Strepsiptera has been associated with Diptera, Siphonaptera, Odonata, Ephemerida, Hymenoptera and Lepidoptera [65]. More recently, four different placements have been suggested as possibilities: membership in the Coleoptera [66], sister group to the Coleoptera [67,68], outside the Holometabola [2] and sister to Diptera [4,69]. Based on molecular studies of 18S rDNA, Whiting et al [4] proposed the grouping of Diptera + Strepsiptera under the name Halteria. Chalwatzis et al [70,71] reached similar conclusions about the relationships between Diptera and Strepsiptera using a larger dataset of 18S rDNA sequences. Later, other authors [72-74] attributed that grouping to an artefact due to LBA, becoming one of the best examples of LBA ever, called "the Strepsiptera problem".

Further phylogenetic evidence using other nuclear data contradicted the Halteria hypothesis, and supported associations between Strepsiptera and Coleoptera. Rokas et al (1999) [75] pointed to an intron insertion in *en *class homeoboxes of Diptera and Lepidoptera but not in Strepsiptera, Coleoptera or Hymenoptera, arguing that an intron loss is an improbable event. Based on a different approach, Hayward et al (2005) [76] used the structure of the USP/RXR hormone receptors, which showed a strong acceleration of evolutionary rate in Diptera and Lepidoptera, to reject the Halteria clade and to provide strong evidence for Mecopterida. Bonneton et al (2003, 2006) [77,78], confirmed the USP/RXR approach of Hayward et al and added the ecdysone receptor (ECR; NR1H1) to the analysis, confirming a Mecopterida monophyletic group. However, these two studies were not able to define clear associations for Strepsiptera. Misof et al (2007) [10] published a large phylogeny of Hexapoda using 18S rDNA and applying mixed DNA/RNA substitution models. Although they recovered well-supported hexapod basal relationships, they obtained very low resolution and unclear relationships within Holometabola.

Recent molecular studies using extensive nuclear data seemed to contradict the Halteria hypothesis again, recovering a close relationship between Coleoptera and Strepsiptera [11,13,14]. First, Wiegmann et al (2009) [11] used a complete dataset of six nuclear protein coding genes including all holometabolan orders. They recovered Coleopterida and provided statistical evidence discarding LBA effects. They found some conflicting signal using individual genes like *cad*, which recovered Halteria, a result that was attributed to LBA because this is a rapidly evolving locus. Longhorn et al (2010) [13] used a total of 27 ribosomal proteins and tested several nucleotide-coding schemes for 22 holometabolan taxa, including two strepsipteran species, where a majority of the schemes tested recovered Coleopterida. McKenna and Farrell (2010) [14] raised identical conclusions using a total of 9 nuclear genes for 34 holometabolan taxa. Also, the Coleopterida have been recently recovered when using large morphological datasets [79,80]. Thus, evidence supporting Coleopterida has grown in recent years, suggesting that the phylogenetic placement of Strepsiptera has been definitely identified.

In summary, classical and most recent morphological and molecular studies based on nuclear data support the Mecopterida and Coleopterida hypotheses. Until now no mitochondrial evidence backed these hypotheses and our results are the first to fully agree with the most generally accepted point of view.

### The Hymenoptera position and the basal splitting events of Holometabola

Depending on algorithm conditions, we observed inconsistencies among analyses in the Hymenoptera position (Figure 2). For example, the fact of using six gamma rate categories instead of four in ML-AA, or simply performing 5000000 instead of 1000000 runs (each with chain stability checked with Tracer) for BI-DNA, or assigning different partitions for DNA, tRNA and rRNA produced alternative results, either ((Diptera + Lepidoptera) Hymenoptera) Coleoptera) or (Diptera + Lepidoptera) + (Coleoptera + Hymenoptera) (not shown). Similar problems when using mitochondrial data have been previously described by Castro and Dowton (2005, 2007) [81,82] regarding this question, namely inconsistencies depending on the ingroup and outgroup selection and the analytical model. Overall, they described a tendency in their analyses to group Hymenoptera as sister taxa to Mecopterida, but they also found Hymenoptera or Hymenoptera + Coleoptera as the most basal lineages in some of their trees.

When using the BI-AA-CAT method our mitochondrial overview suggests a sister relationship of Hymenoptera with Mecopterida, placing Coleopterida outside a clade comprising the other examined holometabolan insects. This result coincides with one of the classical morphological points of view [3,83,84], some nuclear evidence [77], and with morphological and nuclear combined analyses [4,5] that recovered Coleoptera at the base of Holometabola (but not Strepsiptera). A phylogeny inferred from 356 anatomical characters by Beutel et al (2010) [80] placed Hymenoptera as the basal holometabolous insects and recovered a paraphyletic Mecopterida, although these groups were not strongly resolved. A morphological study based on characters of the thorax contributed by Friedrich and Beutel (2010) [79] offered two scenarios depending on the phylogenetic algorithm used: Coleopterida as the most basal group in the Bayesian analysis, but Hymeoptera as the most basal when using parsimony. Several hypotheses based on morphology situate hymenopterans as sister to Mecopterida [85,86], grouping coleopterans with the basal Endopterygota [see references in [87]], or with Neuroptera (not present in our dataset) [2,3,5,84-90]. Also, based on the analysis of wing characters, Kukalova-Peck & Lawrence (1993) [68] proposed an alternative phylogenetic hypothesis consisting in a most basal position for the Hymenoptera. Such discrepancies enhance the view that morphological characters are rather useless in order to determine the phylogenetic position of Hymenoptera within the Holometabola [69].

Our results do not support the most recent molecular studies based on nuclear data, all of them reporting Hymenoptera as the most basal holometabolan insects, for example, the phylogenomic results contributed by Savard et al (2006) [9] using a total of 185 nuclear genes. Since these authors were using emerging genome projects to assemble and analyze all the genes, they only were able to use 8 taxa with 4 orders of holometabolan insects represented (Diptera, Lepidoptera, Coleoptera and Hymenoptera). Their phylogeny resulted in a supported Coleoptera sister to Mecopterida clade, leaving Hymenoptera at the base. Zdobnov and Boork (2007) [8] obtained the same conclusions in another phylogenomic approach, using 2302 single copy orthologous genes for 12 genomes representing the same 4 holometabolous insect groups. Based on a dataset with similarly limited taxon sampling, and using the gain of introns close to older pre-existing ones as phylogenetic markers, Krauss et al (2008) [12] arrived to the same conclusion identifying 22 shared derived intron positions of Coleoptera with Mecopterida, in contrast to none of Hymenoptera with Mecopterida. Additionally, phylogenies with a large number of markers and a complete taxon sampling also gave rise to the same conclusions [11,13,14]. Therefore, mitochondrial data under the CAT model avoids obviously wrong relationships caused by LBA and recovers one of the two main current hypotheses. This hypothesis has been proposed mostly based on morphological evidence and differs from most recent nuclear and genomic results. This issue remains thus an open question deserving deeper study.

### Paraneoptera phylogeny and the position of Phthiraptera

**Figure 3:**
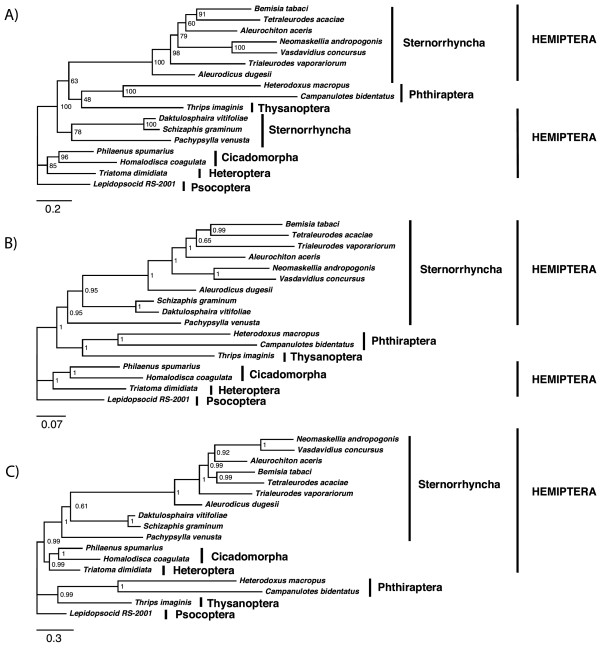
**Paraneoptera phylogenies**. Paraneoptera phylogeny using A) ML-AA (3501 positions), B) BI-DNA (10202 positions) and C) BI-AA-CAT (3501 positions). Values at nodes show bootstrap or posterior probabilities, and scale bar represents substitutions/site.

For Paraneoptera we observed once more an array of topological changes depending on the method used. In ML-AA (Figure 3A), Sternorrhyncha was recovered as paraphyletic with respect to Phthiraptera and Thysanoptera, which evidences the tendency of the method to join lineages according to relative branch length. Indeed, the white flies clade (Sternorrhyncha: Aleyrodoidea) displays a faster substitution rate than their relatives *Daktulosphaira vitifoliae*, *Schizapis graminum *and *Pachypsylla venusta*, and it seems to attract other long-branched clades: Phthiraptera and Thysanoptera. Using BI-DNA (Figure 3B), the topology improved and grouped all Sternorrhyncha representatives, although a paraphyletic Hemiptera remained. Only when using BI-AA-CAT (Figure 3C) a topology with most long-branched taxa not clustered and with a monophyletic Hemiptera was recovered.

Classically, Hemiptera is divided in two suborders: Homoptera and Heteroptera. Homoptera includes Sternorrhyncha and Auchenorrhyncha (Cicadomorpha + Fulgoromorpha). However, according to inferred phylogenies from 18S rDNA, Euhemiptera (Heteropterodea (Heteroptera + Coleorrhyncha) + Auchenorrhyncha) were proposed as sister group of Sternorrhyncha, leaving Homoptera as paraphyletic [91-93], which is currently the most accepted hypothesis. Our mitochondrial analyses with BI-AA-CAT produced the same conclusions as 18S rDNA datasets. Thus, Euhemiptera was recovered as a robust clade formed by Cicadomorpha plus Fulgoromorpha (a group known as Auchenorrhyncha) plus Heteropterodea, while Homoptera (Sternorrhyncha + Cicadomorpha + Coleorrhyncha) was paraphyletic with respect to the heteropteran *Triatoma dimidiata*, which appeared in all the tested methods as sister to Cicadomorpha with high support. In this study we were not able to test the Auchenorrhyncha paraphyly due to the lack of a Fulgoromorpha genome when the analyses were performed.

A sistergroup relationship between the Hemiptera and Thysanoptera, jointly known as Condylognatha [94,95], has been proposed based on morphological characters and supported by 18S rDNA data [96]. Moreover, the closest relatives of this group seem to be the Psocodea (= 'Psocoptera' + Phthiraptera). Although Homoptera paraphyly is fully accepted, at molecular level it has just been tested with nuclear single-gene phylogenies and the full Paraneoptera has never been studied with mitochondrial genomes. There is a broad acceptance that Paraneoptera is a monophyletic group of hemimetabolous insects, comprising the Hemiptera, Thysanoptera, and Psocodea, but the basal relationships within this group are quite controversial. The Condylognatha proposal (Hemiptera + Thysanoptera) was supported by several studies [83,97-102], although spermatological characters [2], fossil studies [103,104] and combined molecular and morphological data [4] suggested an alternative sister-group relationship between Psocodea and Thysanoptera. Psocodea, however, is a fully accepted clade, even if the two orders included have been proposed to be mutually paraphyletic [96,105].

In our results, even using the AA-BI-CAT, which seemed to eliminate LBA artefacts for other clades, the basal Paraneoptera relationships were in contradiction with the generally accepted hypotheses. Psocodea was not monophyletic because Thysanoptera was recovered as the closest to Phthiraptera, and consequently Condylognatha is not supported. In fact, *Thrips imaginis *and the Phthiraptera genomes, were recovered as sister with high support in most of the methods tested. This result, although unexpected, cannot be readily dismissed as wrong and deserves more scrutiny (see for example [106]).

### Eumetabola: Assessing the limits of the mitogenomic data

**Figure 4:**
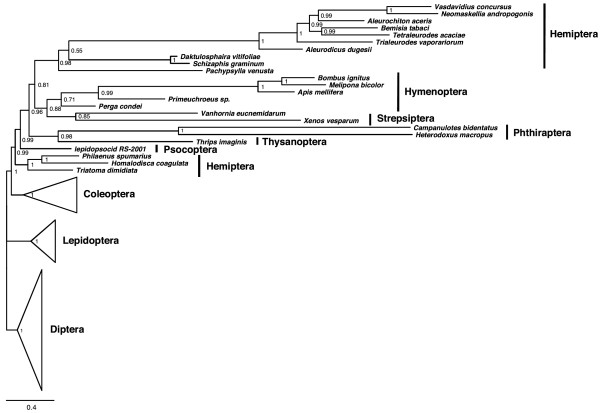
**Eumetabola phylogeny**. Eumetabola phylogeny obtained using BI-AA-CAT (3288 positions). Values at nodes show posterior probabilities and scale bar represents substitutions/site.

To try to understand what are the informative limits of the insect mitochondrial genomes, we raised the global divergence in our dataset by joining Paraneoptera and Holometabola genomes. With ML-AA and BI-DNA, all long branches grouped, a result obviously produced by LBA. Although resolution improved when the BI-AA-CAT was used, this method was not able to deal with the increased divergence and the result was not satisfactory (Figure 4). A tree with similar problems resulted when using the model CAT-BP, optimized to reduce the effects of compositional heterogeneity. Mainly, the hymenopterans remained within the long-branched cluster, although successfully including the short-branched Hymenoptera *Perga condei *with their relatives. Generally, although some signal was detected, this must be lower than the noise and considerable systematically erroneous relationships were recovered.

Given the observed inconsistencies when the divergence is increased in insects, we should question the utility of the Arthropoda mitogenomes to recover supra-ordinal phylogenetic information because of mutational saturation, at least with the current methodological offer.

Mitogenomic data have been used to successfully address several phylogenetic questions within mammals [107,108] and birds [53]. In both cases, however, relationships at the root level were not fully resolved, like the basal relationships between paleognaths and neognaths in birds, and Theria (marsupials plus placentals) versus Marsupionta (monotremes plus marsupials) hypotheses in mammals. In an ecdysozoan mitogenomics study testing the affinities of the three Panarthropoda phyla and the Mandibulata vs. Myriochelata hypothesis, Rota-Stabelli et al (2010) [60] also described difficulties caused by LBA. They obtained reasonable results only by removing rapidly evolving lineages and appyling the CAT model. Within Arthropoda, Nardi et al (2003) [22] presented an unexpected result based on a mitogenomic phylogeny: the paraphyly of the hexapods. They found crustaceans as sister to Insecta, and Collembola as sister to both. This result was discarded by Delsuc et al (2003) [23], who tried to avoid saturation and composition heterogeneity by recoding nucleotides as purines (R) and pyrimidines (Y), recovering then a monophyletic Hexapoda. Later, Cameron et al (2004) [21] performed a detailed battery of analysis including the major arthropod groups to test the hexapod monophyly. They removed hymenopteran and paraneopteran genomes from the analysis due to their extreme divergences. Even so, they could not obtain a conclusion about the relationships due to the strong topological instability of the trees.

Cook et al (2005) [24] assessed the same question concluding that Crustacea and Hexapoda were mutually paraphyletic, although not including unstable lineages like Hymenoptera, the wallaby louse (*Heterodoxus macropus*) and others with long branches. Removing some important lineages, may strongly affect the general topology and the inferred evolutionary history. Carapelli et al (2007) [25] obtained the same conclusions by Cook et al (2005) [24] when including several additional genomes in the analysis and using a new model of amino acid replacement for Pancustracea, MtPan. They presented two large phylogenies based on DNA and protein alignments that supported a non-monophyletic Hexapoda, both obtaining a higher likelihood score under MtPan than when using MtArt or MtRev. However Carapelli et al did not test as many strategies and combinations like others did, in which case they would have probably arrived to similar contradicting conclusions. Some of the relationships within the Insecta they recovered were in strong disagreement with previous morphological and molecular evidence. For example: 1) They recovered a supported association between Strepsiptera and the Crustacean *Armillifer armillatus *(Pentastomida), two problematic yet clearly not related organisms sharing exceptional rates of evolution. 2) Diptera was included in the polyneopteran insect lineage when using BI-DNA and as an independent lineage from all the rest of the Insecta class when using BI-AA with MtPan model. 3) The positions of the orthropterans *Gryllotalpa orientalis *and *Locusta migratoria *remained unclear in their analysis. 4) The plecopteran *Pteronarcys *was recovered outside the polyneopterans, clustering with the Diptera. All of these cases were strongly supported by Bayesian posterior probabilities, but it is known that biases in deep phylogenies might increase supports of incorrect relationships. They attributed the non-monophyletic clade of Holometabola to a biased sampling, lacking orders like Mecoptera, Siphonaptera, Trichoptera or Neuroptera, but once more they removed the Hymenoptera from the analyses. Timmermans et al (2007) [26] re-evaluated the Collembola position using ribosomal protein gene sequences, which resulted in the supported monophyly of Hexapoda for all methodologies used (MP, ML and BI). They also found the inconsistency between nuclear and mitochondrial data when analyzing pancustracean relationships and clearly claimed that "caution is needed when applying mitochondrial markers in deep phylogeny".

The limitations of the mitochondrial genome as phylogenetic marker were already pointed out by Curole and Kocher (1999) [109], when an increasing number of mitogenomes were sequenced and resulting phylogenies conflicted with morphological and nuclear hypotheses in the deep relationships of tetrapods and arthropods, as well as in mammals [110]. Within Insecta, more than one hundred mitogenomes are available now in GenBank/DDBJ/EMBL and they have been used to successfully resolve intra-ordinal relationships, such as in Diptera [28], Hymenoptera [29], Orthoptera [30] and Nepomorpha (Heteroptera) [31]. We report the difficulties to work on inter-ordinal relationships within Insecta, although showing that they can be generally avoided by using the BI-AA under the site-heterogeneous mixture model (CAT). However, we conclude that divergences in mitochondrial sequences above super-order levels represent an insurmountable problem for current methods. This result is at least valid for Arthropoda mitochondrial genomes, but difficult to extrapolate to other groups of organisms. We must remember some exceptional characteristics of the Insecta and Arthropoda in general, like high AT-content, the parasitic life-styles present in some groups or explosive radiation events in others. It is thus possible that a more relaxed evolutionary process in other metazoans allows for slightly deeper studies, and the limits of each dataset should be independently assessed.

### Mitochondrial single genes reliability

Gene exclusion is one of the commonly used strategies to improve phylogenies and we tried to better understand the contribution of each mitochondrial gene to the phylogeny. Indeed, we found important variability in the phylogenetic signal of the different genes (Table 1). Five of the thirteen genes were especially informative in the topology resolution: *cox1*, *nad1*, *cytb*, *nad2 *and *nad4*. On the contrary *atp6*, *atp8*, *cox2*, *cox3 *and *nad4L *datasets produced the most different topologies. According to scale-factor values, *nad1*, *nad3*, *nad4*, *nad5 *and *atp6 *were the genes with a global divergence closest to the whole mitochondrial genome. *nad2*, *nad6*, *cox1*, *atp8 *and *cytb *were the outliers in this case, giving the most deviated values. For both parameters, only *nad1 *and *nad4 *were among the best genes. Interestingly, some of the genes, for example *cox1*, performed very well regarding topology, but strongly deviated in divergence. The opposite applies to *atp6*. The unusually fast substitution rate of *cox1 *compared to the mitochondrial mean (scale-factor = 2.0732) should be highlighted because this is the most common mitochondrial marker in single gene studies of insects and it is broadly used to infer molecular clocks in evolutionary time-based studies. According to this result, *cox1 *seems to be a highly variable gene in insects, which makes it very suitable for the study of recent relationships and for DNA barcoding studies of this group of organisms.

**Table 1:** Scale-factor and Robinson-Foulds distances for individual mitochondrial genes.

	Scale-factor	Robinson-Foulds
***nad1***	**1.1089**	**4**

***nad2***	0.7142	**5**

*nad3*	**0.9596**	7

***nad4***	**0.9771**	**6**

*nad5*	**0.9095**	8

*nad6*	0.7075	8

*nad4L*	0.7822	10

***cox1***	2.0732	**2**

*cox2*	0.8565	8

*cox3*	1.1510	8

*atp6*	**1.0279**	9

*atp8*	0.6826	11

***cytb***	1.3414	**4**

The best values for each parameter and the five best-scored genes for Robinson-Foulds are highlighted in bold.

Considering topology resolution as a priority in systematic studies, we selected the five best-scored genes for further comparisons with the whole genome. The phylogeny that resulted from their combined use reproduced a very similar topology to that of the entire dataset in several cases (Table 2). Thus, the resolution of the five gene combinations is comparable to that of a full genome, a result that could be explained by the inclusion of noise by the less informative genes. In conclusion, we suggest that the use of a selection of the most suitable genes is a valid (and simpler) strategy that produces results equivalent to the use of the entire genome. In order to apply this strategy in insect mitochondrial studies, we identify *cox1*, *nad1*, *cytb*, *nad2 *and *nad4 *as the best genes for topology, and *nad1*, *nad3*, *nad4*, *nad5 *and *atp6 *for branch lengths. We emphasize the importance of deciding what aspect of the mitogenome we want to estimate using a subset of genes, whether topology or branch length, because some of these genes (notoriously *cox1*) perform very well in one regard and poorly in the other.

**Table 2:** Scale-factor and Robinson-Foulds distance when comparing five concatenated genes versus whole genome in different datasets.

	Scale-factor	Robinson-Foulds
Paraneoptera	1.19471	1

Paraneoptera (long-branched taxa excluded)	1.03562	1

Holometabola	1.26578	7

Holometabola (long-branched taxa excluded)	0.58196	2

Eumetabola	0.79679	13

Eumetabola (long-branched taxa excluded)	1.10733	4

## Conclusions

Although several innovative phylogenetic methods have been developed to improve mitochondrial phylogenetic trees in some groups of organisms, results have been controversial in insects, leading to different conclusions that most often disagree with more generally accepted relationships obtained from nuclear and morphological data. Thus, insects constitute a perfect model to test different methodologies and to better understand phylogenetic inference behaviour. Here we tested a battery of those strategies with three datasets of complete mitochondrial genomes of Insecta, including problematic taxa usually excluded from the analyses, and we compared the results with the current nuclear and morphological state of knowledge. The results suggested that the use of amino acid sequences instead of DNA is more appropriated at the inter-ordinal level and that the use of the site-heterogeneous mixture model (CAT) under a Bayesian framework, currently implemented in the software PhyloBayes, substantially avoids LBA artefacts. We show that inferring phylogenies above the super-order level constitutes the limit of the phylogenetic signal contained in insect mitochondrial genomes for currently available phylogenetic methods. For many of the relationships studied, we demonstrate for the first time that, with the proper methodology, mitochondrial data supports the most generally accepted hypotheses based on nuclear and morphological data. Thus, we confirm the non-monophyly of Homoptera within Paraneoptera, and recover Strepsiptera as a sister order to Coleoptera. In the basal splitting events in Holometabola we recover the Hymenoptera-Mecopterida association, and Coleoptera + Strepsiptera form a clade sister to the rest of Holometabola, which coincides with one of the two most accepted hypotheses. Recovered basal relationships in Paraneoptera differ from the currently accepted hypothesis in the position of Phthiraptera, which is recovered as sister to Thysanoptera, resulting in a paraphyletic Psocodea. By comparing single-gene to whole genome tree topologies, we select the five genes best performing for deep Insect phylogenetic inference. The combined used of these five genes (*cox1*, *nad1*, *cytb*, *nad2 *and *nad4*) produces results comparable to those of mitogenomes, and we recommend the prioritary use of these markers in future studies.

## Methods

### Alignments

A total of 55 complete or almost complete Eumetabola mitochondrial genomes (17 of Paraneoptera and 38 of Holometabola) were downloaded from GenBank (Additional file 1: Table S1). Analyses were conducted using 3 datasets; 1) Holometabola, 2) Paraneoptera and 3) Eumetabola (Paraneoptera + Holometabola) in order to assess phylogenetic behaviour in a higher divergence level.

Every gene was translated to protein according to the arthropod mitochondrial genetic code and individually aligned using Mafft 5.861 [111]. To produce the DNA alignments, gaps generated in the protein alignment were transferred to the non-aligned DNA sequences using PutGaps software [112]. The resulting DNA and protein alignments for each gene were concatenated after removing problematic regions using Gblocks 0.91 [113] under a relaxed approach [15] with the next set of parameters: "Minimum Number Of Sequences For A Conserved Position" = 9, "Minimum Number Of Sequences For A Flank Position" = 13, "Maximum Number Of Contiguous Nonconserved Positions" = 8, "Minimum Lenght Of A Block" = 10, "Allowed Gap Positions" = "With Half", and the kind of data was "by codons" for DNA and "Protein" for the aminoacids.

tRNA and rRNA sequences were individually aligned using ProbconsRNA 1.1 [114] and ambiguously aligned regions removed with Gblocks with the same parameters used for DNA. For the Paraneoptera dataset, both *tRNA-Leu *sequences from *Aleurodicus dugesii *were removed because they were extremely long in comparison to the rest and affected the alignment mechanism. For the Holometabola dataset, the large subunit ribosomal RNA sequence from *Anophophora glabripennis*, the *tRNA-Met *from *Ostrinia nubilalis *and *Ostrinia furnacalis*, and the *tRNA-Trp *from *Cysitomia duplonata *were unusually short and were not included. All these fragments were excluded from the Eumetabola dataset as well. Sequences were concatenated, and gaps were used instead of the removed RNAs and the few lacking coding genes.

### Strategies for phylogenetic analysis

We tested several strategies for phylogenetic analyses on the three datasets. These differed in the phylogenetic algorithm, the treatment of saturation, and the use of different models of replacement: 1) Maximum likelihood on protein alignments under the MtRev and MtArt models (ML-AA); 2) Maximum likelihood on protein alignments under Empirical profile mixture models (20 and 60 profiles) (ML-AA-CAT) [38,115] 3) Bayesian inference on protein alignments under the MtRev model; 4) Bayesian inference on DNA alignments including only first and second codon positions for the 13 coding genes under the GTR+I+G model (BI-DNA); 5) Bayesian inference on DNA alignments including first and second codon positions of the 13 coding genes, plus 22 tRNA and 2 rRNA [35] and under the GTR+I+G model; 6) Bayesian inference with a site specific rate model for all DNA + RNA positions [35] 7) Bayesian inference under the CAT model on DNA alignments including only first and second codon positions of the 13 coding genes; 8) Bayesian inference under the CAT model of protein alignments from the 13 coding genes (BI-AA-CAT) (Additional file 1: Table S2).

For maximum likelihood analyses, the software PhyML 2.4.4 [116] with the empirical MtRev model and six gamma rate categories was used. PhyML-CAT applying mixture models (C20 and C60) [115] was used when testing an alternative to empirical rate matrices in ML. For Bayesian inference, we used MrBayes v. 3.1.2 [117] and PhyloBayes 2.3 [38]. For MrBayes calculations in DNA alignments we used two partitions (first and second position of every codon), the GTR+I+G model, and four chains of 5.000.000 trees, sampling every 5000 generations. When including coding genes + tRNA + rRNA, sequences were partitioned in three independent partitions, one for each sequence type. For MrBayes analyses on protein alignments we used the MtRev model and four chains of 1.000.000 trees, sampling every 1000 generations, and applied a burn-in of 10% generations. For PhyloBayes analyses we used the site-heterogeneous mixture model CAT model for aminoacid sequences and the GTR-CAT model for the nucleotide sequences, and we run two independent chains of 5000 cycles, removing the first 1000 and sampling one point every five. For the site-specific rate model, characters were divided into six discrete rate categories using TreePuzzle [118] and partitioned in MrBayes from fastest to slowest, following a similar approach than in Kjer & Honeycutt [35]. Convergence of independent runs was checked with the software Tracer v1.4.

For the whole Eumetabola dataset, the CAT-BP model was tested, using the software nhPhylobayes v.023 [119,120]. This model is supposed to better account for amino-acid compositional heterogeneity, because it allows breakpoints along the branches of the phylogeny at which the amino acid composition can change. The number of components in the mixture were fixed to 120, according to the previous CAT-based phylogeny for Eumetabola. Four independent chains were run, and only two of them converged after highly demanding computation. Taking every tenth sampled tree, a 50% majority rule conseus tree was computed using the converged chains.

To statistically compare the CAT model with the stardard site-homogeneus models, cross validation statistics with PhyloBayes 3.3b were performed between the amino acid models (MtRev and CAT), as described in Philippe et al (2011) [121].

### Mitochondrial single genes reliability

In order to explore the contribution of each individual gene to the concatenated tree, 13 single-gene phylogenies from the Paraneoptera dataset were reconstructed with BI-DNA excluding third codon positions. We scored each single-gene resulting phylogeny based on Robinson-Foulds distances and relative scale-factor values [122] using the complete mitochondrial tree as reference. The 5 best-scored genes were selected according to Robinson-Foulds distances and they were used to infer Paraneoptera, Holometabola and Eumetabola 5-gene phylogenies. Again, Robinson-Foulds distances and relative scale-factor values were calculated. In the same way, we also tested 5-gene performance when following a common practice in mitochondrial phylogenies of insects: the removal of rapidly evolving lineages with branch lengths deviating from the mean of the reference tree. To do that, taxa with a divergence to the root of the tree higher than 0.5 substitutions/position for Paraneoptera and Holometabola datasets and higher than 0.6 substitutions/position for the Eumetabola were removed. Thus, a total of 6 datasets were scored for the Robinson-Foulds distance and the scale-factor.

## Authors' contributions

GT designed the experiments and analyzed the data. GT and RV contributed in discussing and writing the paper. Both authors read and approved the final manuscript.

## Supplementary Material

Additional file 1 **Additional Text, Figures and Tables**. a) Table S1. List of mitochondrial genomes used in the study. b) Table S2. Number of characters in the final alignments for each phylogenetic reconstruction method tested. c) Simulations methods. d) Simulations results and discussion. d) Figure S1. Simulations. e) References. Click here for file
