# Supplementary material for: What is the phylogenetic signal limit from mitogenomes? The reconciliation between mitochondrial and nuclear data in the Insecta class phylogeny
Source: BMC Evol Biol. 2011 Oct 27;11:315. doi: 10.1186/1471-2148-11-315 (PMC3213125; doi:10.1186/1471-2148-11-315)
Supplement: Additional file 1 — Additional Text, Figures and Tables. a) Table S1. List of mitochondrial genomes used in the study. b) Table S2. Number of characters in the final alignments for each phylogenetic reconstruction method tested. c) Simulations methods. d) Simulations results and discussion. d) Figure S1. Simulations. e) References. [file 1471-2148-11-315-S1.PDF]

Additional file

What is the phylogenetic signal limit from mitogenomes? The reconciliation between mitochondrial and nuclear data in the Insecta Class phylogeny

Gerard Talavera & Roger Vila

Table S1 - List of mitochondrial genomes used in this study

Organism	GenBank Code	Taxonomical group
**Holometabola**		
Drosophila simulans	NC_005781.1	Diptera
Drosophila sechellia	NC_005780.1	Diptera
Drosophila mauritiana	NC_005779.1	Diptera
Drosophila melanogaster	NC_001709.1	Diptera
Drosophila yakuba	NC_001322.1	Diptera
Chrysomya putoria	AF352790.1	Diptera
Cochliomyia hominivorax	NC_002660.1	Diptera
Haematobia irritans	NC_007102.1	Diptera
Dermatobia hominis	NC_006378.1	Diptera
Bactrocera dorsalis	NC_008748.1	Diptera
Bactrocera oleae	NC_005333.1	Diptera
Ceratitis capitata	NC_000857.1	Diptera
Simosyrphus grandicornis	NC_008754.1	Diptera
Tricophtalma punctata	NC_008755.1	Diptera
Cydistomyia duplonata	NC_008756.1	Diptera
Anopheles gambiae	NC_002084.1	Diptera
Anopheles quadrimaculatus	NC_000875.1	Diptera
Aedes albopictus	NC_006817.1	Diptera
Bombyx mandarina	NC_003395.1	Lepidoptera
Bombyx mori	NC_002355.1	Lepidoptera
Antheraea pernyi	AY242996.1	Lepidoptera
Coreana raphaelis	DQ102703.1	Lepidoptera
Ostrinia furnacalis	NC_003368.1	Lepidoptera
Ostrinia nubilalis	NC_003367.1	Lepidoptera
Adoxophyes honmai	DQ073916.1	Lepidoptera
Bombus ignitus	DQ870926.1	Hymenoptera
Melipona bicolor	NC_004529.1	Hymenoptera
Apis mellifera	NC_001566.1	Hymenoptera
Vanhornia eucnemidarum	NC_008323.1	Hymenoptera
Primeuchroeus sp.	DQ302102.1<br>DQ302101.1	Hymenoptera
Perga condei	AY787816.1	Hymenoptera
Anoplophora glabripennis	NC_008221	Coleoptera
Crioceris duodecimpunctata	NC_003372.1	Coleoptera
Tribolium castaneum	NC_003081.1	Coleoptera
Rhagophthalmus lufengensis	DQ888607.1	Coleoptera
Rhagophthalmus ohbai	AB267275.1	Coleoptera
Pyrocoelia rufa	NC_003970.1	Coleoptera

Xenos vesparum	DQ364229.1	Strepsiptera
**Paraneoptera**		
Neomaskellia andropogonis	NC_006159.1	Hemiptera
Vasdavidius concursus	AY648941.2	Hemiptera
Aleurochiton aceris	NC_006160.1	Hemiptera
Bemisia tabaci	NC_006279.1	Hemiptera
Tetraleurodes acaciae	NC_006292.1	Hemiptera
Trialeurodes vaporariorum	NC_006280.1	Hemiptera
Aleurodicus dugesii	NC_005939.1	Hemiptera
Daktulosphaira vitifoliae	DQ021446.1	Hemiptera
Schizaphis graminum	NC_006158.1	Hemiptera
Pachypsylla venusta	AY278317.1	Hemiptera
Philaenus spumarius	AY630340.1	Hemiptera
Homalodisca coagulata	AY875213.1	Hemiptera
Triatoma dimidiata	NC_002609.1	Hemiptera
Heterodoxus macropus	NC_002651.1	Phthiraptera
Campanulotes bidentatus	NC_007884.1	Phthiraptera
Thrips imaginis	NC_004371.1	Thysanoptera
Lepidopsocid RS-2001	NC_004816.1	Psocoptera

Table S2 - Number of characters in the final alignments for each phylogenetic reconstruction method tested. Resulting trees shown in figures 2-4 are indicated.

	Paraneoptera	Holometabola	Eumetabola
ML (Protein)	2731 *(Fig.3)*	3501 *(Fig.2)*	3288
BI (Protein)	2731	3501	3288
BI (DNA 1st and 2nd position)	7010	7368	7232
BI (DNA 1st and 2nd position + RNA)	9536 *(Fig.3)*	10202 *(Fig.2)*	9548
BI (DNA + RNA) - Site specific rate model	13068	13889	-
BI (DNA 1st and 2nd position) - CAT model	7010	7368	7232
BI (Protein) - CAT model	2731 *(Fig.3)*	3501 *(Fig.2)*	3288 *(Fig.4)*

Simulations methods

We compared the efficiency of the protein-based phylogenetic reconstruction strategies using simulations. We performed simulated protein alignments with 1000 amino acid positions conducted along a reference phylogenetic tree with eight tips, with a global divergence equivalent to the Holometabola BI-DNA tree. In order to imitate possible LBA effects, two unrelated branches were forced to be six times longer than the average length of the rest (in the Holometabola BI-DNA tree the longest branches were four times bigger). One hundred simulations were performed using Seq-Gen [1] with six categories of rate heterogeneity (alfa = 0.872) and the MtRev evolutionary model. From these simulations, maximum likelihood trees with six categories of rate heterogeneity were

inferred with Phyml 2.4.4, Bayesian inference with Mr.Bayes 3.1.2 and MtRev model, and PhyloBayes 2.3 under the CAT model. After that, we calculated the scale-factor, a relative value for comparing branch lengths between two trees, and the Robinson-Foulds distance, which calculates the topological differences between two trees, using Ktreedist 1.0 software [2] from each resulting tree versus the reference tree used to conduct the simulations.

Simulations results and discussion

Simulations were performed to confirm the ability of the CAT model to suppress the LBA bias compared to ML-AA and BI-DNA. We created two unrelated long branches in a tree with eight terminals with a similar divergence to the Holometabola dataset, and also exaggerated this divergence 2 and 3 times. The general tendency of the simulation test results was the same than the one observed in real data. For the three divergence levels explored, BI-AA-CAT produced the lowest percentage of trees grouping the two long branches as sister taxa. In divergence x1, BI-CAT did not group the long branches in any of the simulations (0%), while we obtained a 9% for ML-AA and a 10% for BI-DNA. For divergence x2, long branches were grouped together in 3% of the cases for BI-AA-CAT, 12% for ML-AA and 26% for BI-DNA. Finally for divergence x3, the values increased to 10% for BI-AA-CAT, 34% for ML-AA and 38 % for BI-DNA. These percentages do not evaluate intermediate LBA effects, where both branches might be closer than they should, but not strictly sisters. To evaluate the topological differences between the simulated trees and the reference topology, presumably a product of LBA, we calculated Robinson-Foulds distances and calculated the average of the 100 simulations for each divergence type and method. This revealed again the better performance of BI-AA-CAT, which obtained the lowest values, followed by ML-AA and BI-DNA respectively (Figure S1). A better performance of the amino acid sequences versus DNA was also reflected in these results, and their use together with a site-heterogeneous mixture model under a Bayesian framework is the suggested combination to avoid LBA artefacts.

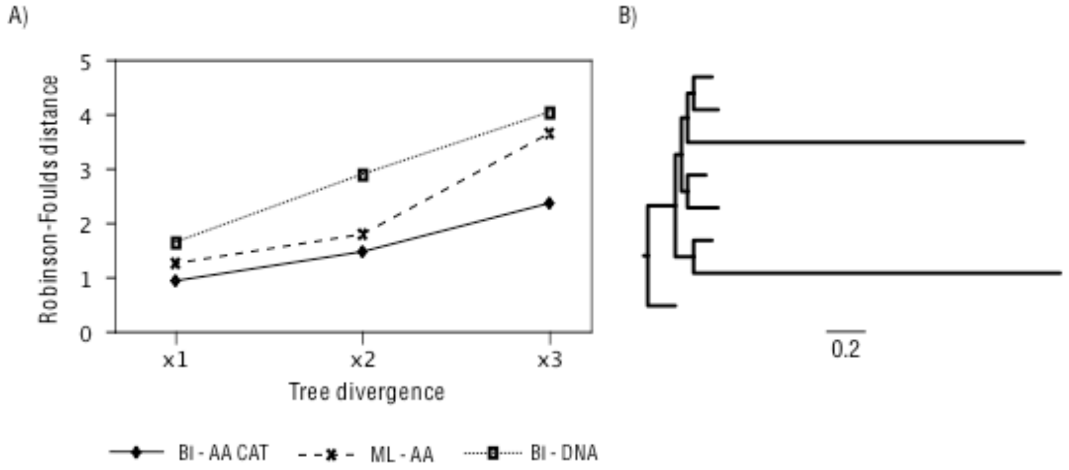

Figure S1 - Simulations

A) Average Robinson-Foulds distances relative to the reference tree calculated with ML - protein sequences (dotted line with squared symbols), BI - DNA excluding third codon positions (dashed line with cross symbols) and BI - protein with CAT model (solid line with diamonds) for three different tree divergences. B) Reference tree (divergence x1) used to conduct simulations. Scale bar represents 0.2 substitutions/site.

References:

1. Rambaut A, Grassly NC: **Seq-Gen: an application for the Monte Carlo simulation of DNA sequence evolution along phylogenetic trees.** *Comput Appl Biosci 1997,* **13**:235-238
2. Soria-Carrasco V, Talavera G, Igea J, Castresana J: **The K tree score: quantification of differences in the relative branch length and topology of phylogenetic trees.** *Bioinformatics 2007,* **23**:2954-2956
